# Comparison of Frictional Properties of CVD-Grown MoS_2_ and Graphene Films under Dry Sliding Conditions

**DOI:** 10.3390/nano9020293

**Published:** 2019-02-19

**Authors:** Dae-Hyun Cho, Jaehyuck Jung, Chan Kim, Jinhwan Lee, Se-Doo Oh, Kwang-Seop Kim, Changgu Lee

**Affiliations:** 1 Department of Mechatronics Engineering, Gyeongnam National University of Science and Technology, 33, Dongjin-ro, Jinju-si, Gyeongsangnam-do 52725, Korea; cho@gntech.ac.kr; 2 SKKU Advanced Institute of Nanotechnology, Sungkyunkwan University, 2066 Seobu-ro, Jangan-gu, Suwon, Gyeonggi 16419, Korea; geljjh@gmail.com; 3 Korea University of Science & Technology (UST), Nanomechatronics, 217 Gajeong-ro, Yuseong-gu, Daejeon 34113, Korea; chankim@kimm.re.kr; 4 Nano-Convergence Mechanical Systems Research Division, Korea Institute of Machinery & Materials (KIMM), 156 Gajeongbuk-ro, Yuseong-gu, Daejeon 34103, Korea; 5 School of Mechanical Engineering, Sungkyunkwan University, 2066 Seobu-ro., Jangan-gu, Suwon, Gyeonggi 16419, Korea; zzazangzzang@gmail.com; 6 Fuel and Emission System R&D Center, Korea Automotive Technology Institute, 303 Pungse-ro, Pungse-myeon, Dongnam-gu, Cheonan, Chungnam 31214, Korea; sdoh@katech.re.kr

**Keywords:** friction, wear, MoS_2_, graphene, Chemical Vapor Deposition

## Abstract

In the present study, dry friction and wear properties of atomically thin CVD-grown graphene and MoS_2_ films on SiO_2_/Si substrates were compared at low (72 MPa) and high (378 MPa) contact pressures. Analysis of atomic force microscopy images of these films verified that the MoS_2_ films, which were directly grown on the SiO_2_/Si substrates, had clean surfaces and made conformal contacts with the substrates. In contrast, the graphene film showed many contaminants on its surface and was loosely bonded with its SiO_2_/Si substrate due to its wet transfer from a Cu foil to the substrate. The MoS_2_ film exhibited friction and wear properties superior to those of the graphene film both at low and high contact pressures. We found that the clean sliding surface and strong bonding with SiO_2_/Si were the main causes of the superiority of the MoS_2_ film compared to the graphene film. Mild wear occurred in a layer-by-layer fashion at low contact pressure for the MoS_2_ film. At high contact pressure, severe wear occurred due to failure at the boundary between the MoS_2_ films and the underlying substrates. At both contact pressures, friction did not increase immediately after the removal of the MoS_2_ film from the SiO_2_/Si substrate because the film transferred onto the counter sliding surface and served as a lubricant.

## 1. Introduction

Solid lubricants have been used to make sliding systems effective. In the past few decades, solid lubricants made up of layered crystalline structures and displaying low-friction properties have been reported. These properties have been ascribed to the low resistance of the atomic shear plane to shear force resulting from weak van der Waals interactions between the neighboring layers [1]. Recently, even single-layer graphene, hexagonal boron nitride and molybdenum disulfide (MoS_2_) have been reported to exhibit low friction when strongly anchored onto an underlying substrate [2,3]. These observations imply that the low shear strength of the atomic shear plane is not the only determinant of the outstanding lubricities of these materials.

Of these layered materials, graphite is the most widely used solid lubricant because of its superb lubricity, low cost, and abundance in nature [4]. As large-area synthesis and transfer of single-layer graphene have become feasible, graphene has been emerging as a promising candidate for serving as an atomically thin solid lubricant [5,6,7]. Atomically thin solid lubricants can be effectively utilized for demanding applications such as microelectromechanical and nanoelectromechanical systems (MEMS and NEMS), and bio-implants, where sliding occurs over small distances, specifically in the sub-micrometer range. Graphene, in particular, has been reported to show excellent tribological performance in micro-scale dry contact [8].

When large-area graphene is synthesized by carrying out chemical vapor deposition (CVD), mainly copper substrates are used as a catalyst in the reaction [9] to utilize graphene as a protective film for a surface, therefore, a transfer of the graphene from the copper substrate to the target surface is necessarily required. A temporary sturdy substrate such as polymethyl methacrylate (PMMA) and polydimethylsiloxane needs to be used to support the highly compliant graphene and hence prevent it from becoming damaged during its transfer. However, a problem in this regard is that several wet chemical steps, which can contaminate the graphene surface, are incorporated in the transfer process [10]. While, as mentioned above, single-layer graphene displays outstanding lubricity when strongly bound to its underlying substrate [3], contaminants that become adsorbed onto the graphene surface during the transfer process can interfere with the strong bonding between the graphene film and the substrate. Therefore, strong bonding between the transferred graphene and its underlying substrate might not be sufficient to produce low friction. Also, when sliding surfaces make a micro-scale contact area, contaminants would have an undesirable effect on the tribological performance of the graphene.

Another issue limiting the application of graphene as a solid lubricant is the difficulty of controlling its thickness over a large area. Among tribology specialists, atomically thin graphene film is considered to be an excellent solid lubricant for MEMS or NEMS applications, but not so much so for typical macro-scale sliding systems. However, in such small-scale systems, controlling film thickness is crucial because the thickness determines the clearance between contacting surfaces. Therefore, an effective way to control the thickness of graphene needs to be developed in order to utilize it as a solid lubricant in MEMS and NEMS applications [7].

MoS_2_ is known as an effective lubricant like graphite and can be directly synthesized on an arbitrary target substrate by reacting a Mo metal source on the substrate with H_2_S gas [11]. The direct synthesis without a transfer process can offer advantages from the tribological perspective. First, it can overcome the contamination problems expected in the case of graphene. Accordingly, CVD-grown MoS_2_ film would be expected to strongly bind its substrate and display excellent intrinsic tribological characteristics in sliding systems. Second, when carrying out such a direct synthesis, it is possible to control the produce a film with uniform thickness over a large area. Therefore, lubrication engineers can control the clearance between the contacting surfaces in MEMS and NEMS applications. In this context, CVD-grown MoS2 would be considered to be an excellent atomically thin solid lubricant for tribological applications if it can be made to have frictional properties surpassing those of CVD-graphene.

Many types of wear mechanisms have been proposed over the past few decades. For example, adhesion, plowing, corrosion, erosion, surface fatigue and seizure have all been proposed for explaining wear [12,13]. A sliding system is rarely dominated by any one wear mechanism, and instead generally more than two wear processes occur simultaneously. Hence, the friction and wear properties of any particular sliding system are very hard to predict. For designers who wish to select a suitable solid lubricant, the wear rate of the sliding surfaces and the life of the sliding system are of particular interest. Generally, they broadly classify the wear of various materials as either ‘mild’ or ‘severe’ [14,15,16]. Mild wear results in a smooth surface and severe wear gives a rough surface with a high wear rate.

In the present study, we compared the dry friction and wear properties of CVD-grown MoS_2_ with various thicknesses to those of CVD-grown graphene at low (72 MPa) and high (378 MPa) contact pressures. These two contact pressures were selected to observe contrasting wear behaviors of MoS_2_ films. SiO_2_/Si was chosen as a substrate for the graphene and MoS_2_ films because it is a widely used material for MEMS/NEMS applications and electronic devices. In our investigation, CVD-grown MoS_2_ effectively reduced the friction and showed better resistance to wear than did CVD-grown graphene. Furthermore, a transition from mild to severe wear of MoS_2_ was observed.

## 2. Materials and Methods

### 2.1. Sample Preparation

MoS_2_ films were directly grown on SiO_2_/Si substrates as reported by Lee et al. [11]. In order to synthesize 2-layer (2L), 4-layer (4L), and 12-layer (12L) MoS_2_ films, 0.5-nm-, 1-nm-, and 3-nm-thick Mo films were, respectively, deposited on the SiO_2_/Si substrates by performing e-beam evaporation under high-vacuum conditions. The Mo-film-coated SiO_2_/Si samples were placed in a quartz vacuum chamber and heated up to 750 °C with Ar flowing at a rate of 100 standard cubic centimeters per minute (sccm). Then, a mixture of H_2_S/H_2_/Ar (1:5:50) gases was passed into the reaction chamber for 15 min under a chamber pressure of 3.1 × 10^−1^ Torr. To enhance the crystallinity of the MoS_2_ films, the samples were annealed at 1000 °C for one hour with a continuous 100 sccm flow of Ar and cooled down to room temperature also with an Ar flow. Generally, each layer of MoS_2_ was about 0.65 nm thick [17]. 

Monolayer (1L) graphene film, which exhibit 0.35 nm in thickness, was grown on a Cu foil (Alfa Aesar, Ward Hill, MA, USA, 0.025 mm thick, 99.8%) using a typical growth process [9,18]. The Cu foil was put into a solution of sulfuric acid (Sigma Aldrich, St. Louis, MO, USA, 95%) and hydrogen peroxide (Sigma Aldrich, 30%) in a 3:1 ratio and rinsed several times with deionized (DI) water. The foil was then loaded in the quartz vacuum chamber and annealed at 1000 °C with a 10 sccm flow of H_2_ gas for 30 min in order to remove surface oxide and to grow grain size of Cu. After the annealing process, graphene was grown by introducing a CH_4_ gas flow of 30 sccm for 30 min and slowly cooled down to room temperature over the course of 40 min. Synthesized graphene films on the Cu foil were transferred to a SiO_2_/Si wafer. To prevent significant damage to the graphene during the liquid transfer process, the graphene on the Cu foil was coated with PMMA. Since graphene grew on both sides of the Cu foil, the graphene film on the opposite side of the coated PMMA was removed by subjecting it to oxygen plasma etching. The Cu foil underneath the graphene was then etched by applying an aqueous solution of 0.1 M ammonium persulfate (Sigma Aldrich, 98%) to the foil for 4 h and rinsing the foil treated this way with DI water several times. Finally, the graphene film with the PMMA coating was scooped from the DI water with a SiO_2_/SI substrate and gradually dried. The resulting composite was baked at 70 °C for 15 min and soaked in acetone overnight in order to remove the PMMA coating.

As mentioned above, SiO_2_/Si was chosen as the substrate for the graphene and MoS_2_ films to compare their friction and wear properties and to be considered for future MEMS/NEMS applications.

**Figure 1:**
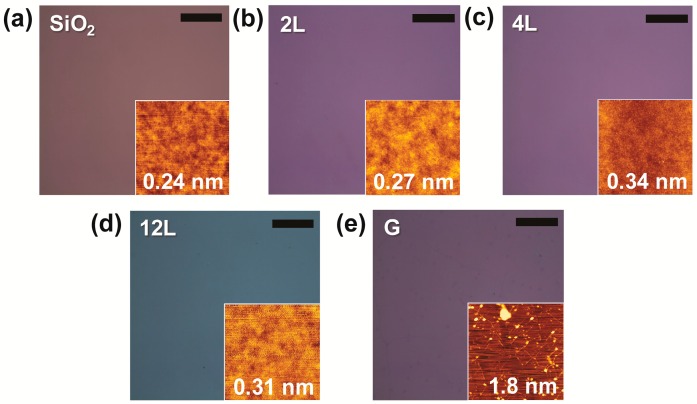
Optical and AFM images (insets, 4 µm × 4 µm scan) of (**a**) a bare SiO_2_/Si sample, and (**b**) 2L MoS_2_, (**c**) 4L MoS_2_, (**d**) 12L MoS_2_, and (**e**) 1L graphene each on a SiO_2_/Si substrate. Texts in the insets indicate RMS surface roughness. Scale bars, 20 µm.

Figure 1 shows optical and AFM images of a bare SiO_2_/Si sample, and of 2L, 4L, 12L MoS_2_ and 1L graphene each on a SiO_2_/Si substrate. Uniform colors were observed in each of these optical images, indicating that the synthesized films each had a uniform thickness. The thickness of each synthesized MoS_2_ film was verified by acquiring and analyzing their Raman spectra. As shown in Supplementary Figure S1a, these spectra showed that the MoS_2_ films were indeed 2L, 4L, and 12L thick, respectively. Supplementary Figure S1b shows the Raman spectrum of 1L graphene. 

We next compared the RMS roughness of the MoS_2_ films on SiO_2_/Si to that of the bare SiO_2_/Si. AFM tapping mode with a sharp Si AFM tip (Budget Sensors, Sofia, Bulgaria, Tap300Al-G, resonant frequency 300 kHz) was employed for this comparison. As shown in Figure 1, the RMS roughness of each of the MoS_2_ films (Figure 1b–d) was less than a nanometer, comparable to that of the bare SiO_2_/Si (Figure 1a). According to Reference [3], the morphology of an atomically thin film can closely follow that of the underlying SiO_2_/Si substrate, and the film and substrate can show comparable RMS roughness values when they make a conformal contact. Therefore, the comparability of the RMS roughness values of the MoS_2_ films to that of the naked SiO_2_/Si substrate may have arisen from a conformal contact made between each film and the substrate. Such formation of a conformal contact was also consistent with gas-state Mo molecules having moved to and solidified directly on the SiO_2_/Si surface during the MoS_2_ synthesis.

As shown in the AFM image of 1L graphene (Figure 1e), contaminants and wrinkles were observed. During the transfer of graphene from the Cu foil to the SiO_2_/Si substrate, contaminants may have become trapped between the graphene film and its substrate or have become attached to the top of the graphene surface. Such contaminants, mainly composed of the methoxy function and the carboxyl function groups, have been reported to originate from PMMA [19,20]. Also, wrinkles can form on the graphene film because of its extremely high compliance [10]. The contaminants and wrinkles on the graphene film apparently contributed to its relatively high RMS roughness value (1.81 nm) compared to that of bare SiO_2_/Si (0.24 nm).

### 2.2. Sliding Tests

Low-pressure friction measurements were taken using a home-built microtribometer that was previously used for determining frictional characteristics of CVD-grown graphene on the micro-scale [8]. A laser-quality fused-silica plano-convex lens with a radius of 12.9 mm remained stationary during the friction measurement and the MoS_2_- and graphene-coated SiO_2_/Si wafer samples displayed reciprocating motion with a stroke of 2 mm. The friction force was calculated from the displacement bending of the cantilever as measured using the laser displacement sensor. The sample was subjected to a normal load of 30 mN and was made to slide at an average speed of 50 μm/s. In this condition, the calculated Hertzian contact pressure and the contact diameter were about 72 MPa and 28 µm for the fused-silica lens on the uncoated SiO_2_/Si. These conditions allowed us to determine the process by which MoS_2_ films developed mild wear. All measurements were taken in the ambient atmosphere (21 ± 1 °C and 33 ± 3% RH). These measurement conditions allowed us to compare the acquired experimental data with previously reported data from CVD-grown graphene films [8].

In order to observe the frictional behaviors and occurrence of severe wear of MoS_2_ films on the SiO_2_/Si substrate, friction measurements under high pressure were taken using a typical ball-on-disk setup. A stationary SiC ball with a diameter of 10.16 mm was made to slide against a rotating silicon wafer. MoS_2_ and graphene films were deposited onto the rotating disk samples. A sliding speed of 10 mm/s and a normal load of 0.84 N were applied to gradually generate wear damage on the MoS_2_ films. The calculated Hertzian contact pressure and the contact diameter were about 378 MPa and 66 µm, respectively, for the SiC ball on the uncoated SiO_2_/Si. In this sliding condition, we could observe as discussed below a transition of the frictional properties of MoS_2_ on SiO_2_/Si due to wear after several tens of sliding cycles. We recorded the number of sliding cycles at which this transition occurred to compare the wear resistance of MoS_2_ and graphene films on SiO_2_/Si. All measurements were taken in the ambient atmosphere (21 ± 1 °C and 35 ± 5% RH).

## 3. Results and Discussion

**Figure 2:**
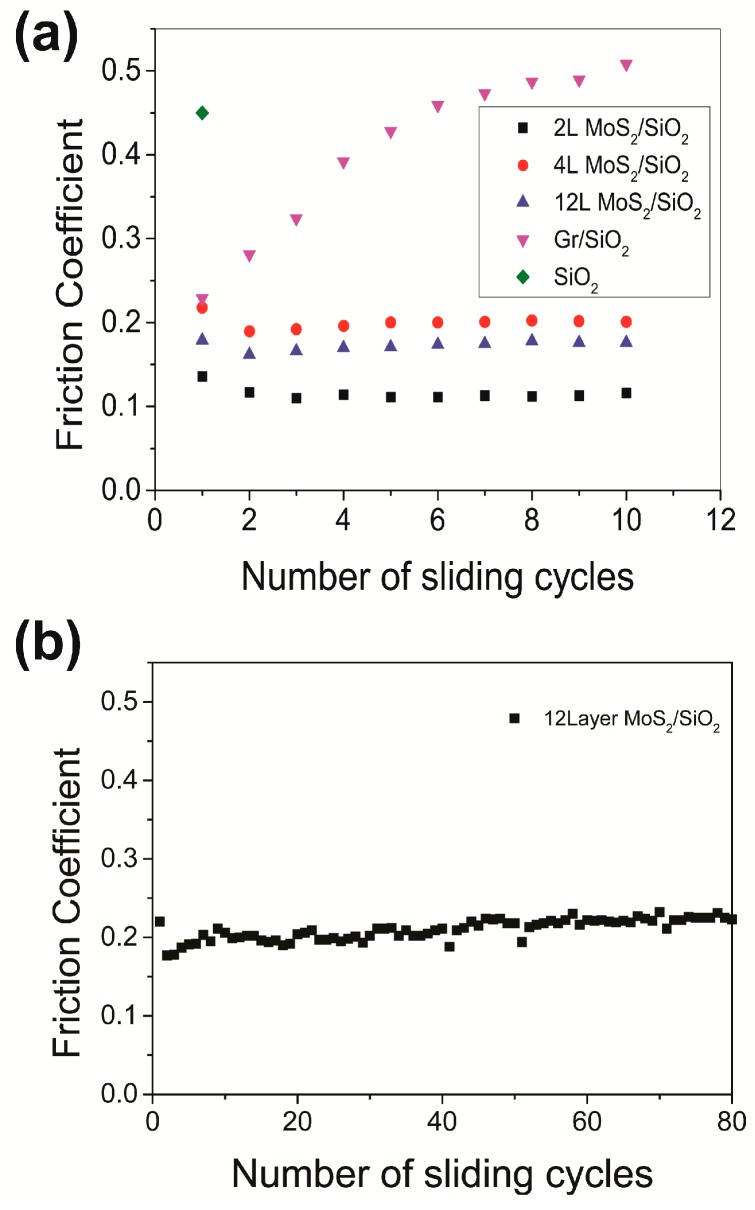
Coefficients of friction determined under low pressure for (**a**) 2L MoS_2_, 4L MoS_2_, 12L MoS_2_, and 1L graphene subjected to up to 10 cycles of sliding and SiO_2_/Si subjected to up to 1 cycle and for (**b**) 12L MoS_2_ subjected to up to 200 cycles of sliding.

Figure 2 shows coefficients of friction of the various samples determined using the low contact pressure. As shown in Figure 2a, friction coefficient for graphene was observed to gradually increase from 0.23 to 0.45 as the number of sliding cycles was increased to ten. The initial value of the coefficient of friction (0.23) was similar to that of graphene on Cu against a fused silica (0.22) reported in Reference [8]. The increased friction coefficient value of 0.45 was comparable to the friction coefficient of the bare SiO_2_/Si substrate. These results taken together indicated that the graphene film was removed and the SiO_2_/Si substrate was exposed by the time ten cycles of sliding were performed.

**Figure 3:**
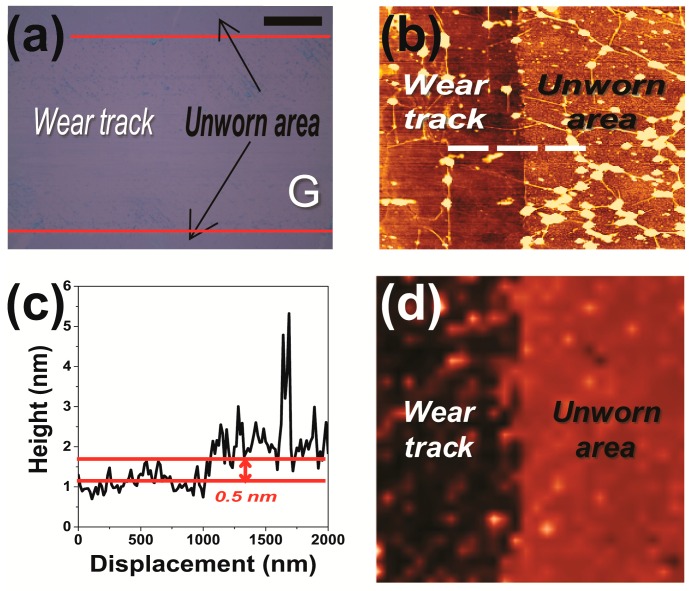
Wear track images of the graphene sample subjected to low pressure and 10 cycles of sliding. (**a**) Optical image (dotted line indicates the boundary between wear track and unworn area), (**b**) AFM image, (**c**) surface height profile along the dotted line in (**b**), and (**d**) Raman spectrum image (2D peak). Black scale bar, 10 µm.

To verify that the graphene film was indeed removed, an optical image of the sample was taken after 10 cycles of sliding as shown in Figure 3a. From the optical contrast between worn and unworn areas, it is possible to define the boundary between worn and unworn areas. For clarity, an AFM image, shown in Figure 3b, was obtained at the boundary between the worn and unworn areas. The difference between the height of the worn area and that of the unworn area was measured to be about 0.5 nm (Figure 3c), corresponding to the thickness of monolayer graphene, and hence indicating that graphene film was removed from its substrate. A Raman spectrum image (2D peak) of the sample was acquired (Figure 3d), and inspection of this image also revealed that the graphene film was removed from the substrate after 10 cycles of sliding, but that small graphene particles remained on the worn track. These results taken together demonstrated the instability of the graphene film exposed to the applied low contact pressure.

Compared to the graphene sample, the tested MoS_2_ samples exhibited lower coefficient of friction values. After 10 cycles of sliding, these values were only 0.1 to 0.2 for the MoS_2_ samples, as shown in Figure 2a. Moreover, the coefficient of friction of the 12L MoS_2_ film remained at a low value of 0.2 even up to 200 cycles of sliding, as shown in Figure 3b. These results verified the superior frictional properties and wear resistance of the MoS_2_ films compared to those of the graphene film. 

**Figure 4:**
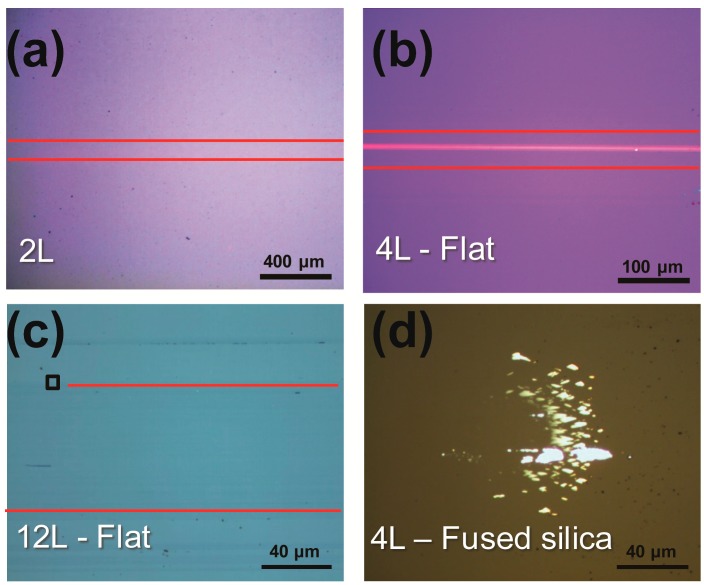
Optical images of the wear tracks of (**a**) 2L MoS_2_, (**b**) 4L MoS_2_, (**c**) 12L MoS_2_, and (**d**) the fused silica counterpart after undergoing 5 cycles of sliding under low pressure. The black box in (**c**) corresponds to the AFM scanned area in Figure 5.

After 10 cycles of sliding, scratch lines on the centers of wear track of each on the 2L and 4L MoS_2_ film can be seen as shown in Figure 4a,b. The color of the scratches is same to the bare SiO_2_/Si substrate. In contrast to theses, blue color remained on the wear track of 12L MoS_2_ sample after the sliding test. This result indicated that the SiO_2_/Si substrate was not exposed completely even after 10 cycles of sliding. Some of the wear debris piled up at the end of the wear track of the coated flat samples and the rest adhered to the counterpart (fused silica ball) as shown in Figure 4d. Although the scratch lines formed and the underlying substrate was exposed for the 2L and 4L MoS_2_ samples, there was no significant change in the coefficient of friction after 5 cycles of sliding. Perhaps the debris transferred to the counter-surface of the fused silica functioned as the tribofilm. Previously, debris transferred from CVD-grown graphene was observed to form the tribofilm and to show low friction, comparable to that of unworn graphene [8]. 

**Figure 5:**
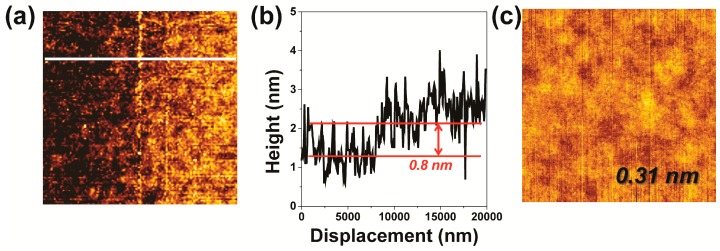
Analysis of the wear track of 12L MoS_2_. (**a**) A 2 μm × 2 μm AFM image obtained from the region of the sample corresponding to the black box shown in Figure 4c. (**b**) Surface height profile of the slice of the sample corresponding to the white line in panel (**a**). (**c**) A 500 nm × 500 nm AFM image obtained from the wear track (white box in Figure 4c). The RMS roughness value of the worn area of the sample is indicated.

Slips can occur at the interlayer of a layered material when a friction force is released parallel to the interlayer plane [1]. If wear occurs in a layer-by-layer fashion, the underlying layer would be exposed to the sliding surface after detachment of the top layer. The newly exposed layer should have a surface morphology essentially identical to that of the original top layer if there is no plastic deformation during sliding, and the wear depth should correspond to integer multiples of the monolayer thickness. To test this hypothesized layer-by-layer wear mechanism, we obtained an AFM image of the boundary between the worn and unworn areas of the 12 L MoS_2_ sample subjected to 5 cycles of sliding, as shown in Figure 5a. The difference between the height of the worn area and that of the unworn area was measured to be about 0.8 nm (Figure 5b), corresponding to the thickness of a monolayer of MoS_2_. Also, the worn area here showed an RMS roughness of 0.31 nm (Figure 5c), close to the RMS roughness values measured for the unworn MoS_2_ films shown in Figure 1b–d. Based on these height difference and RMS roughness results, we concluded that the wear of the tested MoS_2_ film occurred in a layer-by-layer fashion.

**Figure 6:**
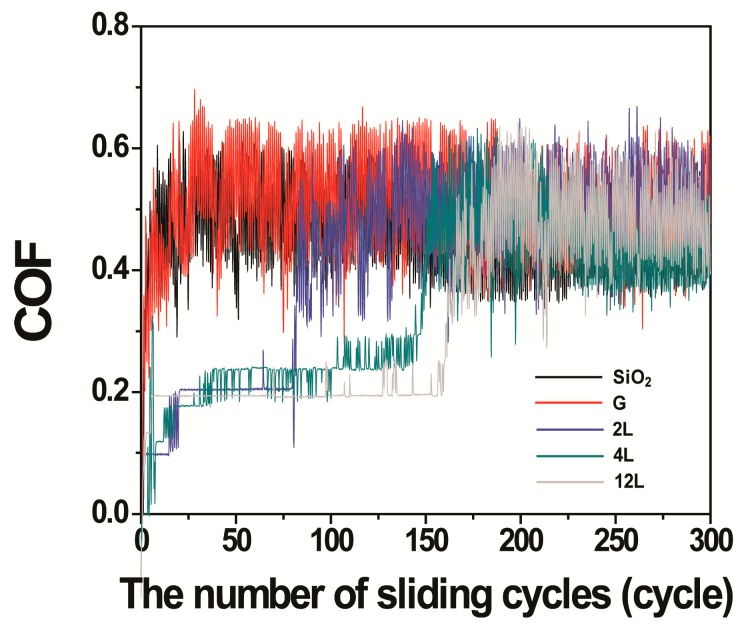
Coefficients of friction (COF) obtained under high pressure from 2L MoS_2_, 4L MoS_2_, 12L MoS_2_, 1L graphene and bare SiO_2_/Si for up to 300 cycles of sliding.

Figure 6 shows coefficients of friction of our various samples obtained under high contact pressure. The measured coefficient of friction of the bare SiO_2_/Si substrate was 0.48 ± 0.07 immediately after the onset of siding and the friction coefficient value of 1L graphene rapidly increased to a very similar value of 0.52 ± 0.08. The similarity of the results for the 1L graphene film and the bare SiO_2_/Si were thought to be caused by the removal of the graphene film and the resulting exposure of the underlying SiO_2_/Si substrate very soon after the sliding was commenced. To test this explanation, we stopped the sliding test only after 20 sliding cycles and inspected the wear track of the 1L graphene sample as shown in Supplementary Figure S2. Severe wear damage on the underlying SiO_2_/Si substrate was observed. This observation demonstrated that the graphene film immediately detached from the SiO_2_/Si substrate under the conditions of high contact pressure.

**Figure 7:**
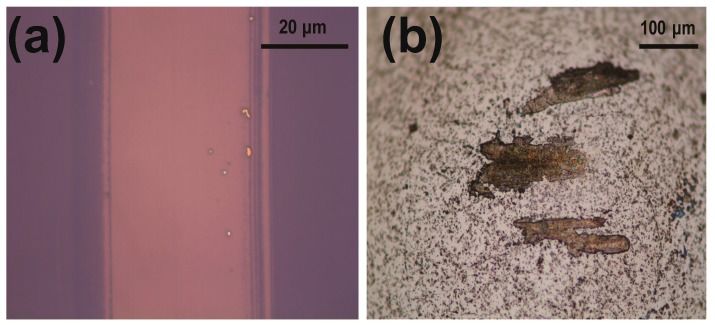
Optical images of the wear track on (**a**) the 2L-MoS_2_-coated flat sample and (**b**) SiC ball after 50 cycles of sliding.

As shown in Figure 6, MoS_2_-coated SiO_2_/Si samples showed relatively low and stable coefficients of friction, in the range 0.18 to 0.24, for the first few dozens of sliding cycles, with the thickest tested MoS_2_ film showing this relatively low friction coefficient for the most cycles. But after several additional cycles, the coefficients of friction did increase, to 0.48 ± 0.05, comparable to the friction coefficient of bare SiO_2_/Si. The eventual similarity of the friction levels of the MoS_2_-coated SiO_2_/Si to that of bare SiO_2_/Si was thought to be caused by the removal of the MoS_2_ film from its substrate. To test this explanation, optical images of the MoS_2_-coated SiO_2_/Si samples were taken after 50 sliding cycles, at which point the coefficient of friction was still low and stable. The optical image of the sample coated with the 2L MoS_2_ film (Figure 7a) showed that the MoS_2_ film was totally removed from its substrate after 50 cycles of sliding, despite the sample having still exhibited the relatively low level of friction, i.e., lower than that of the bare SiO_2_/Si. After the sliding test, the film appeared to have transferred onto the counter-surface of the SiC ball, according to the image of the ball shown in Figure 7b. These results taken together suggested that the transferred MoS_2_ functioned as a solid lubricant on the ball to retain the lower friction level for a few additional cycles of sliding [4,8].

**Figure 8:**
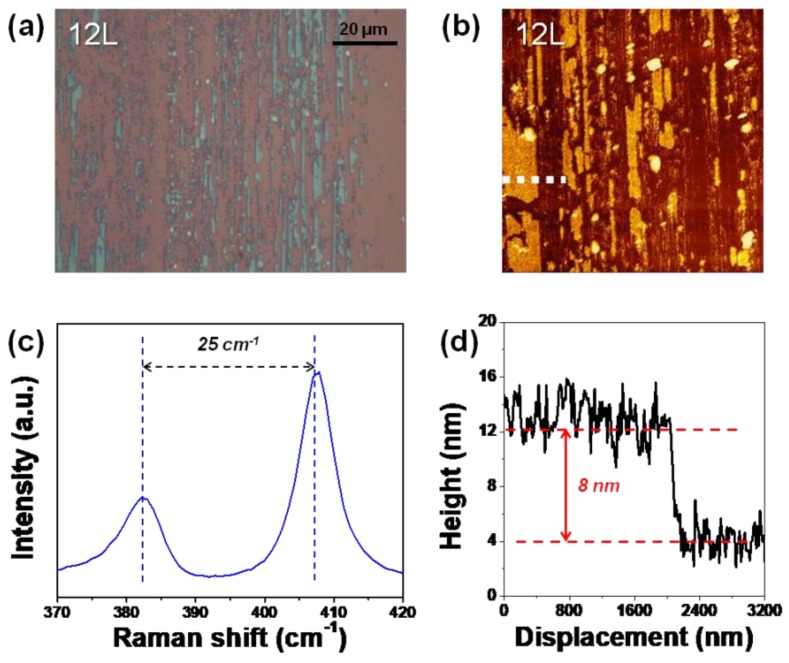
Analysis of the wear track on 12L MoS_2_ formed under high contact pressure after 50 cycles of sliding. (**a**) Optical image and (**b**) 20 μm × 20 μm AFM image of the wear track on 12L MoS_2_. (**c**) Raman spectrum taken from 12L MoS_2_ residue. (**d**) Surface height profile acquired from the dotted line in (**b**).

Inspection of optical and AFM images of the 12L MoS_2_ sample under high contact pressure after 50 cycles of sliding (Figure 8a,b) suggested that residues of the MoS_2_ film remained on its substrate after this sliding. The E_2g_ (left peak in Figure 8c) and A_1g_ (right peak in Figure 8c) Raman signals obtained from these residues verified that they were composed of MoS_2_. The heights of the MoS_2_ residues were measured using tapping mode AFM as shown in Figure 8d, and found to be about 8 nm, closely corresponding to the thickness of 12L MoS_2_. Both the MoS_2_ residues and the exposed SiO_2_/Si surfaces showed nanoscale variations in height. Also, the exposed SiO_2_/Si surfaces did not show significant damage. Based on these results, we speculated that the wear under high contact pressure occurred as a result of the repeated frictional stresses that exceeded the limit of the bonding strength between the 12L MoS_2_ films and the underlying substrates. Cracks at the boundary between the films and the underlying substrates may have formed and propagated, resulting in a chipping off of the wear fragments. Such a wear process is totally different than the layer-by-layer wear mode observed under conditions of low contact pressure. 

## 4. Conclusions

In this study, we compared the friction and wear properties of 2L, 4L, and 12L CVD-grown MoS_2_ to those of the CVD-grown graphene at low (72 MPa) and high (378 MPa) contact pressures. We found that, regardless of thickness, the MoS_2_ films showed lower friction than did the graphene film both at low and high contact pressures. We derived two explanations for the relatively low friction levels of the MoS_2_ films we produced compared to that of the graphene film: One being that the MoS_2_ films exhibited a clean surface because they were synthesized directly on SiO_2_/Si substrates without requiring a transfer process, in contrast to contaminants on the graphene film formed during its transfer process having weakened its lubricity; and the other being the conformal contact between MoS_2_ and the SiO_2_/Si substrates due to the direct growth of the MoS_2_ films on SiO_2_/Si.

The MoS_2_ films also showed resistance to wear superior to that of the graphene film both at low and high contact pressures. At low contact pressure, graphene was rapidly removed from the SiO_2_/Si substrate but MoS_2_ was not. It was observed that the MoS_2_ film occurred in a layer-by-layer fashion and the friction did not immediately increase after removal of the MoS_2_ film. Perhaps the MoS_2_ film transferred to the counterpart during wear and then functioned as a tribofilm.

At high contact pressure, the friction of the sample coated with the MoS_2_ film did not increase immediately after the MoS_2_ film fully detached from its substrate. Similarly, in the condition of low contact pressure, MoS_2_ film transferred to the counterpart served as a lubricant. However, at high contact pressure, the friction of the graphene-coated sample showed an immediate increase after the onset of sliding. A layer-by-layer wear process did not occur on the MoS_2_ films, in contrast to observations in the low contact pressure tests. Instead, wear fragments were generated due to the failure at the boundary between the MoS_2_ films and the underlying substrates.

We concluded that MoS_2_ films have superior potential for reducing friction and wear compared to graphene films. Further tribological studies need to be carried out under various sliding conditions, e.g., loads, speeds, temperatures, and counter materials, because different modes of wear were indicated in the current work for the different loading conditions. Our results can be used for constructing a wear-mechanism map of MoS_2_ films, which would be a helpful design guide for lubrication engineers in the NEMS/MEMS fields. 

## Supplementary Materials

The following are available online at https://www.mdpi.com/2079-4991/9/2/293/s1, Figure S1: Raman spectra of (a) MoS_2_ and (b) graphene samples. The thickness of prepared MoS_2_ samples corresponds to 2L, 4L, and 12L thickness. Graphene corresponds to 1L thickness, Figure S2: Optical images taken from the bare SiO_2_ and 1L graphene samples after 20 sliding cycles on macro-scale. After 20 sliding cycles, sever wear damages were observed on 1L graphene samples. This indicates that the tested 1L graphene cannot protect sliding surfaces.
