# Supplementary material for: Comparison of Frictional Properties of CVD-Grown MoS2 and Graphene Films under Dry Sliding Conditions
Source: Nanomaterials (Basel). 2019 Feb 19;9(2):293. doi: 10.3390/nano9020293 (PMC6410133; doi:10.3390/nano9020293)
Supplement: Supplementary file 1 [file nanomaterials-09-00293-s001.pdf]

nanomaterials

MDPI

Supplementary Information

Comparison of Frictional Properties of CVD-Grown MoS_2 and Graphene Films under Dry Sliding Conditions

Dae-Hyun Cho [1,†], Jaehyuck Jung [2,†], Chan Kim [3,4], Jinhwan Lee [5], Se-Doo Oh [6], Kwang-Seop Kim [3,4,*] and Changgu Lee [2,5,*]

1 Department of Mechatronics Engineering, Gyeongnam National University of Science and Technology, 33, Dongjin-ro, Jinju-si, Gyeongsangnam-do 52725, Korea; cho@gntech.ac.kr
2 SKKU Advanced Institute of Nanotechnology, Sungkyunkwan University, 2066 Seobu-ro, Jangan-gu, Suwon, Gyeonggi 16419, Korea; geljjh@gmail.com
3 Korea University of Science & Technology (UST), Nanomechatronics, 217 Gajeong-ro, Yuseong-gu, Daejeon 34113, Korea; chankim@kimm.re.kr
4 Nano-Convergence Mechanical Systems Research Division, Korea Institute of Machinery & Materials (KIMM), 156 Gajeongbuk-ro, Yuseong-gu, Daejeon 34103, Korea
5 School of Mechanical Engineering, Sungkyunkwan University, 2066 Seobu-ro., Jangan-gu, Suwon, Gyeonggi 16419 , Korea; zzazangzzang@gmail.com
6 Fuel and Emission System R&D Center, Korea Automotive Technology Institute, 303 Pungse-ro, Pungse-myeon, Dongnam-gu, Cheonan, Chungnam 31214, Korea; sdoh@katech.re.kr
* Correspondence: kskim@kimm.re.kr (K.-S.K.); peterlee@skku.edu (C.L.); Tel.: +82-42-868-7700 (K.-S.K.); +82-31-299-4844 (C.L.)
† These authors contributed equally to this work.

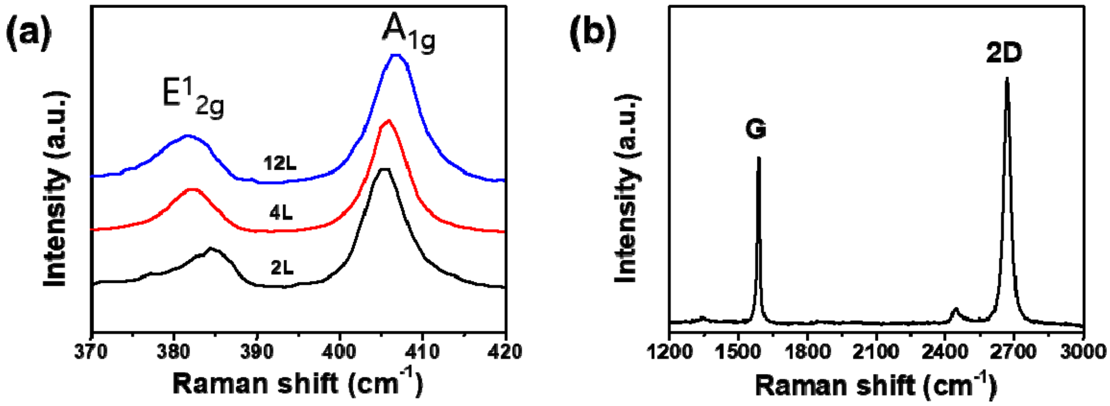

Figure S1. Raman spectra of (**a**) MoS_2 and (**b**) graphene samples. The thickness of prepared MoS_2 samples corresponds to 2L, 4L, and 12L thickness. Graphene corresponds to 1L thickness.

Depending on the number of MoS_2 layer, E^1_{2g} and A_{1g} Raman mode changes. With increasing the number of layer, the frequency of E^1_{2g} exhibits redshifts, while that of A_{1g} shows blueshifts. Therefore, the number of layer (or the thickness) can be determined. C. Lee et al. (Anomalous Lattice Vibrations of Single- and Few-Layer MoS_2, ACS Nano Vol. 4, pp 2695–2700, 2010) proposed the Raman frequencies as an indicator of the layer thickness and this method have been widely used. The frequency differences (cm^{-1}) between these two Raman modes in Supplementary Figure S1 (2L(20), 4L(24), 12L(25)) are very close to the reference data. Also, similar results can be found in another Reference (Y. Lee et. al. Synthesis of Wafer-Scale Uniform Molybdenum Disulfide Films

with Control Over the Layer Number Using a Gas Phase Sulfur Precursor, Nanoscale 2014, 6, 2821–2826.).

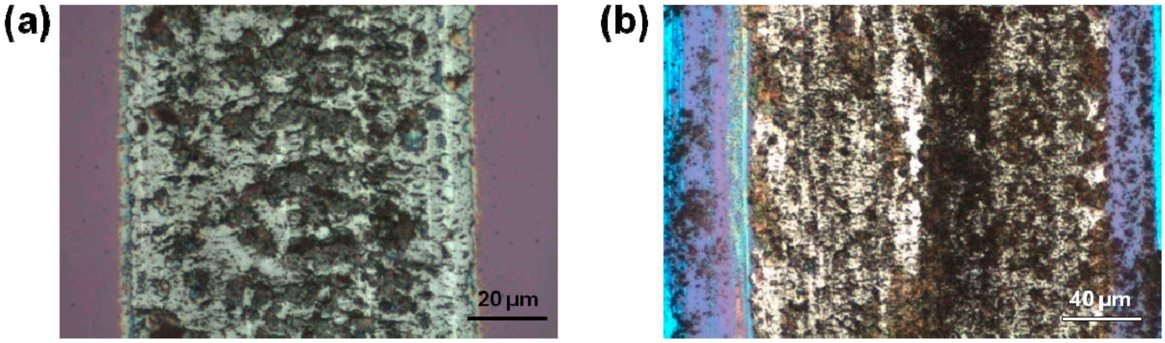

Figure S2. Optical images taken from the bare SiO_2 and 1L graphene samples after 20 sliding cycles on macro-scale. After 20 sliding cycles, sever wear damages were observed on 1L graphene samples. This indicates that the tested 1L graphene cannot protect sliding surfaces.
